# Prostate deformation during hypofractionated radiotherapy: an analysis of implanted fiducial marker displacement

**DOI:** 10.1186/s13014-021-01958-4

**Published:** 2021-12-07

**Authors:** Lukas Knybel, Jakub Cvek, Tomas Blazek, Andrea Binarova, Tereza Parackova, Kamila Resova

**Affiliations:** Department of Oncology, University Hospital Ostrava, 17. listopadu 1790, 708 52 Ostrava, Czech Republic

**Keywords:** Prostate, Deformation, Rigid body, Stereotactic body radiotherapy

## Abstract

**Background:**

To report prostate deformation during treatment, based on an analysis of fiducial marker positional differences in a large sample.

**Material and methods:**

This study included 144 patients treated with prostate stereotactic body radiation therapy after implantation in each of 4 gold fiducial markers (FMs), which were located and numbered consistently. The center of mass of the FMs was recorded for every pair of X-ray images taken during treatment. The distance between each pair of fiducials in the live X-ray images is calculated and compared with the respective distances as determined in the CT volume. The RBE is the difference between these distances. Mean RBE and intrafraction and interfraction RBE were evaluated. The intrafraction and intefraction RBE variability were defined as the standard deviation, respectively, of all RBE during 1 treatment fraction and of the mean daily RBE over the whole treatment course.

**Results:**

We analyzed 720 treatment fractions comprising 24,453 orthogonal X-ray image acquisitions. We observed a trend to higher RBE related to FM4 (apex) during treatment. The fiducial marker in the prostate apex could not be used in 16% of observations, in which RBE was > 2.5 mm. The mean RBEavg was 0.93 ± 0.39 mm (range 0.32–1.79 mm) over the 5 fractions. The RBEavg was significantly lower for the first and second fraction compared with the others (*P* < .001). The interfraction variability of RBEavg was 0.26 ± 0.16 mm (range 0.04–0.74 mm). The mean intrafraction variability of all FMs was 0.45 ± 0.25 mm. The highest Pearson correlation coefficient was observed between FM2 and FM3 (middle left and right prostate) (R = 0.78; *P* < .001). Every combination with FM4 yielded lower coefficients (range 0.66–0.71; *P* < .001), indicating different deformation of the prostate apex.

**Conclusions:**

Ideally, prostate deformation is generally small, but it is very sensitive to rectal and bladder filling. We observed RBE up to 11.3 mm. The overall correlation between FMs was affected by shifts of individual fiducials, indicating that the prostate is not a “rigid” organ. Systematic change of RBE average between subsequent fractions indicates a systematic change in prostate shape.

## Background

Higher doses of external beam radiotherapy improve biochemical failure-free survival in patients with localized prostate cancer [1, 2]. Hypofractionated radiotherapy offers radiobiological advantages and shorter treatment courses but brings with it an even greater need for smaller safety margins to protect surrounding critical structures. Reduction of the planning safety margins requires precise dose delivery based on image guidance. Moreover, the higher dose per fraction can lead to longer treatment times and an increased probability of intrafraction motion. Therefore, understanding patterns of prostate motion and deformation may help to provide more robust and safe treatment in relationship to the organ at risk.

Several studies have described prostate translations and rotations during treatment [3–8]. Various methods have been developed to monitor and compensate intrafraction translations and rotations of the prostate. However, prostate deformation can be hidden in its shifts and rotations and so underestimated. Several studies have evaluated prostate deformation as changes in the prostate surface [9–12] or monitored intermarker distances [13] within the prostate. All these studies have compared pretreatment data with limited data acquired during treatment sessions (3 CT scans [9], randomly assigned fraction [10], once every 5 fraction [11], 8–12 repeat CT scans [12], or a pair of X-ray images before each fraction [13]). Some results of available studies are contradictory: Nichol et al. [10] reported prostate deformation unrelated to bladder and bowel filling, while Kerkhof et al. [14] showed the impact of rectal filling on prostate deformation.

At our institution, the CyberKnife (Accuray, Sunnyvale, CA) is used for prostate hypofractional treatment, with online tracking of implanted gold fiducial markers (FMs) [15]. The positions of FMs are monitored frequently throughout the treatment. Data from online tracking could provide detailed insight into prostate deformation during treatment. In the present study, we reported prostate deformation during treatment based on an analysis of positional differences in fiducial markers in a large sample.

## Material and methods

### Patients

This study included 144 patients treated with prostate stereotactic body radiation therapy (3625 cGy in 5 fractions). Each patient had 4 gold fiducial markers implanted 4 weeks before the treatment to allow any possible edema to resolve and to leave sufficient time for FM fixation in the prostate tissue. FMs were implanted transrectally under ultrasound guidance into the apex, base, and middle left and right lobes of the prostate. There was a minimum distance of 2 cm between FMs, which had to have at least 1 cm of separation on orthogonal imaging, and the angle between the different groups of FMs was > 15° [16, 17]. All patients selected in this study met these criteria what ensures fiducial extraction algorithm to compute rotations accurately. We used the same procedure to locate and mark FMs in the planning CT scan in all patients: the marker in the base was always FM1, and the marker in the apex was always FM4. Moreover, we used the automatic function in the planning system to properly choose the center of mass (CoM) of each FM with the length of 3 mm. Therefore their positions are known in the digitally reconstructed radiographs (DRRs).

Patients were instructed to empty their rectum and drink 0.5 L of water 1 h before the planning CT scan and before each fraction.

### Treatment data analysis

The CyberKnife delivers highly conformal radiation to the prostate, with 6D correction of intrafraction prostate motion, and accuracy < 1 mm [15–18]. Before the treatment, a set of orthogonal X-ray images is acquired and the system determines the position of FMs within the prostate. The image registration is based on alignment of the known DRR FMs positions with the marker locations extracted from the treatment X-ray images [16, 19]. The system automatically calculates the 3D translation and rotation of the target. The first alignment is done by moving the treatment couch until all shifts are below 1 mm and 2°. Additional X-ray images are acquired continuously during treatment, with user-defined frequency. In our department, images are taken each 60 s and the imaging frequency is increased if the target exhibits frequent greater excursions. During treatment, the robotic arm carrying the linear accelerator adjusts the beam position to compensate for residual target displacement. The larger the displacement, the greater is the uncertainty in the accuracy of the robotic correction [20]. For these automatic corrections, we keep the threshold of 1.5 mm for translations and thresholds of 2°, 5°, and 3° for roll, pitch, and yaw rotations, respectively. When a shift exceeds a threshold, beam delivery is paused and the patient is readjusted with couch movement.

The treatment algorithm records the CoM of implanted FMs for every pair of X-ray images taken during treatment (alignment and delivery). The distance between each pair of fiducials in the live X-ray images is calculated and compared with the respective distances as determined in the CT volume. The RBE is the difference between these distances. The fiducial that is contributing the most to the error is marked in the user interface. The system has predefined RBE threshold of 1.5 mm. The default value ensures that the targeting error due to rigid body deformation is below 1 mm. Higher RBE value can represent a local target deformation. If a higher value is accepted, the calculated rotations do not reflect reality and may then cause a geometric miss [21]. All patients included in this study met this threshold before the start of the first fraction, ensuring that no FM shifted excessively in the period between planning CT and the start of the treatment. Additionally, after each X-ray acquisition a separate log-file containing the average rigid body error (RBEavg) in mm and rigid body error of all separate implanted FMs (RBEf1–4) is generated and stored in the system (1 X-ray acquisition = 1 file). The RBE of separate FM at a timestamp is defined as a distance of a fiducial from its corresponding CT position after the system determines the best translation and rotation transformation by a rigid registration of live X-ray images and DRRs. In an ideal case in which there is no deformation, the RBE should be zero [20]. We developed an in-house application which automatically sorts separate log-files containing RBE values according to their time stamps.

To be able to determine the accuracy of calculating FM positions (fiducial localization error), we evaluated log-files with RBE values from the treatment simulations, using an anthropomorphic phantom containing 6 FMs implanted in the rigid cube with required geometry (a minimum distance of 2 cm between FMs, the angle between the different groups of FMs > 15°). The average RBE was 0.14 ± 0.15 mm.

### Motion analysis

Mean RBE and intrafraction and interfraction RBE were evaluated. The intrafraction RBE variability was defined as the standard deviation of all RBE during 1 treatment fraction. Interfraction RBE variability was defined as the standard deviation of the mean daily RBE over the whole treatment course. The system always generates the average rigid body error (RBEavg) and RBEs of all separate implanted FMs; for simplicity, in cases where RBEavg is sufficiently representative, we present only RBEavg results.

### Statistical analysis

Statistical analysis was performed using STATISTICA 13 software (StatSoft, Tulsa, OK). All quantitative data were expressed as mean and SD. Box plots were constructed to visually compare samples. The Pearson correlation coefficient was used to show correlation between FM positions. Repeated-measures ANOVA with Scheffe post-hoc tests was used to evaluate the change in RBE over time (between treatment fractions). All tests were performed at the 5% level of significance.

## Results

A total of 720 treatment fractions with 24,453 X-ray image acquisitions were analyzed. Log-files related to images from patient alignment were not included in the study. FM4 has fewer values of RBE (20,540) compared with the other markers because it had to be disabled for tracking purposes in several cases due to a high RBE value indicating marker migration relative to the reference position.

We observed a quantitatively higher RBE related to FM4 (implanted in the prostate apex) during treatment whose frequency increased with increasing fractions. From 144 patients, only 1 case (0.7%) had a higher RBE of FM4 during the first fraction. As the RBE was under the threshold 1.5 mm at the start of the treatment, we do not think the migration of this marker occurred between the planning CT and the first fraction. We have an internal rule of disabling any FM for tracking if its RBE is constantly above 2.5 mm. During the second fraction, FM4 had to be disabled in 10 patients (7%), and subsequently in 15 (10%), 16 (11%), and 21 (15%) patients for the third, fourth, and fifth fraction, respectively. None of the disabled FM4 could be used again later, indicating a permanent change of position.

**Fig. 1:**
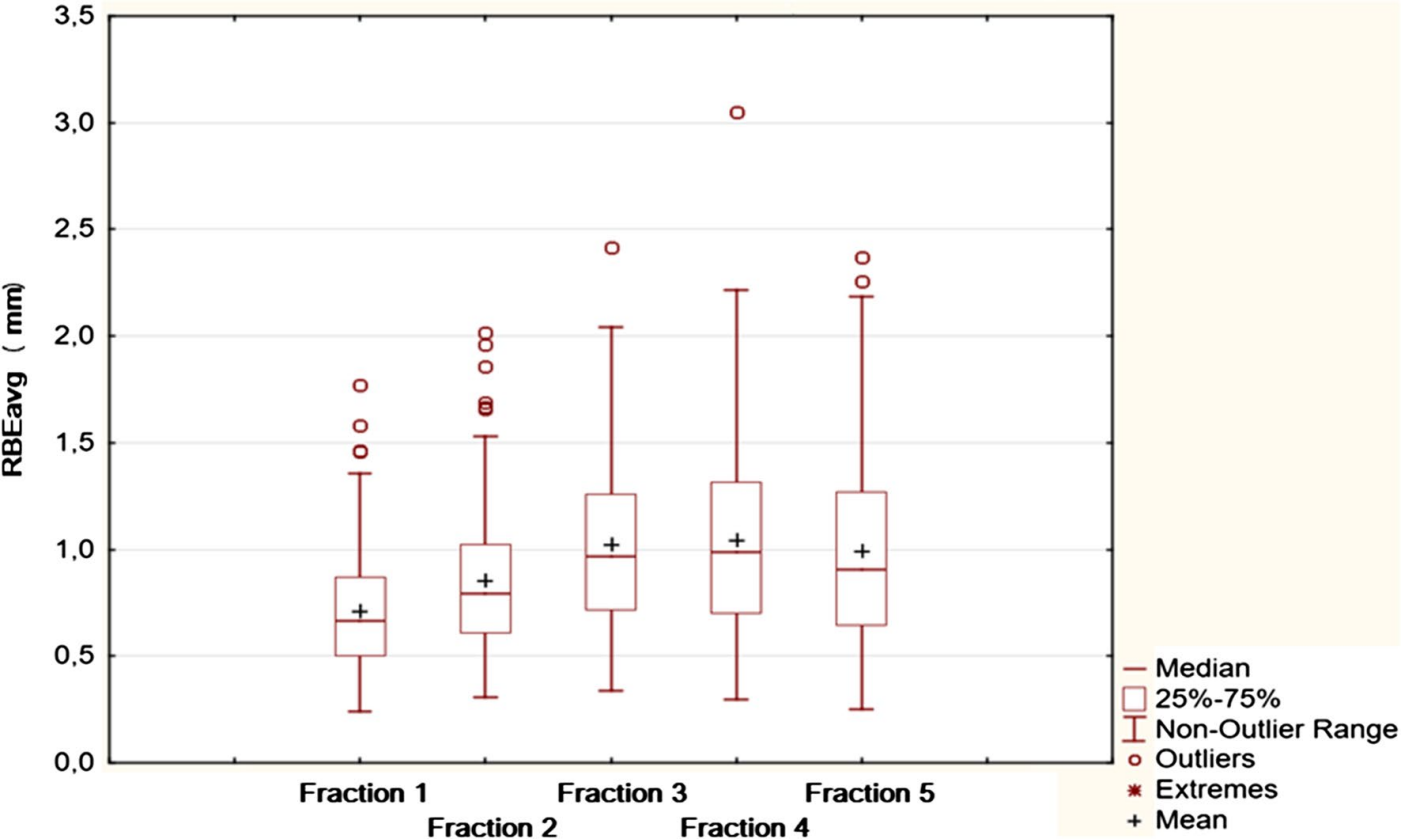
Box plot visualization of RBEavg (mm) for treatment fractions 1–5. Values for fractions 4 and 5 are underestimated due to lower number of observations of FM4 for RBE > 2.5

The mean RBEavg was 0.93 ± 0.39 mm (range 0.32–1.79 mm) over the 5 fractions. The RBEavg was significantly lower for the first and second fractions compared with the others (*P* < 0.001, repeated-measures ANOVA) (Fig. 1). Moreover, values for fractions 4 and 5 are underestimated due to lower number of observations of FM4 for RBE > 2.5. The maximum observed RBE was 11.3 mm, for FM3.

The interfraction variability of RBEavg was 0.26 ± 0.16 mm (range 0.04–0.74 mm). The mean intrafraction variability of all FMs was 0.45 ± 0.25 mm, and no significant difference between fractions was found.

**Fig. 2:**
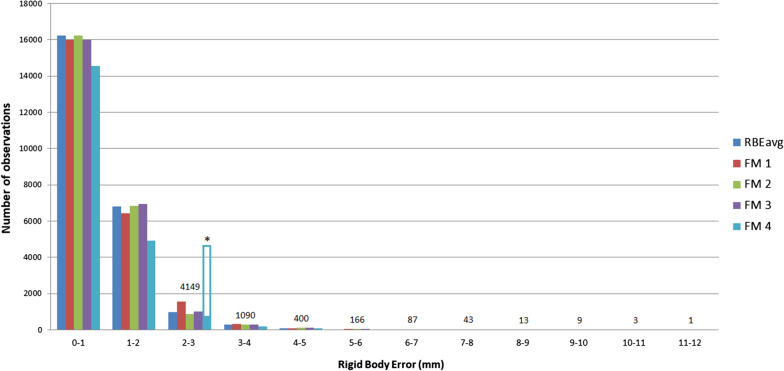
Numbers of observations for FM separately and RBEavg for all cases over the whole treatment. *Visualization of 16% missing values (compared with the other FMs) because FM4 was not used for tracking due to its RBE being consistently > 2.5 mm. The visualization simplifies the situation so that missing values are only given in the range 2–3 mm. Numbers above the columns for values > 2 show the sum of occurrences for FM1-4

RBE values were low in the majority of observations. Figure 2 shows number of observations of RBE values for each FM separately and RBEavg for all cases over the whole treatment. All cases exceeded the RBE threshold of 1.5 mm during the treatment course. Values above the threshold caused treatment interruption. Typical practice at our department is to wait 1 min and then try to acquire another image. If the problem with high RBE persists, the patient is asked to try to empty their rectum, drink 0.5 L of water, and wait approximately 30 min.

**Fig. 3:**
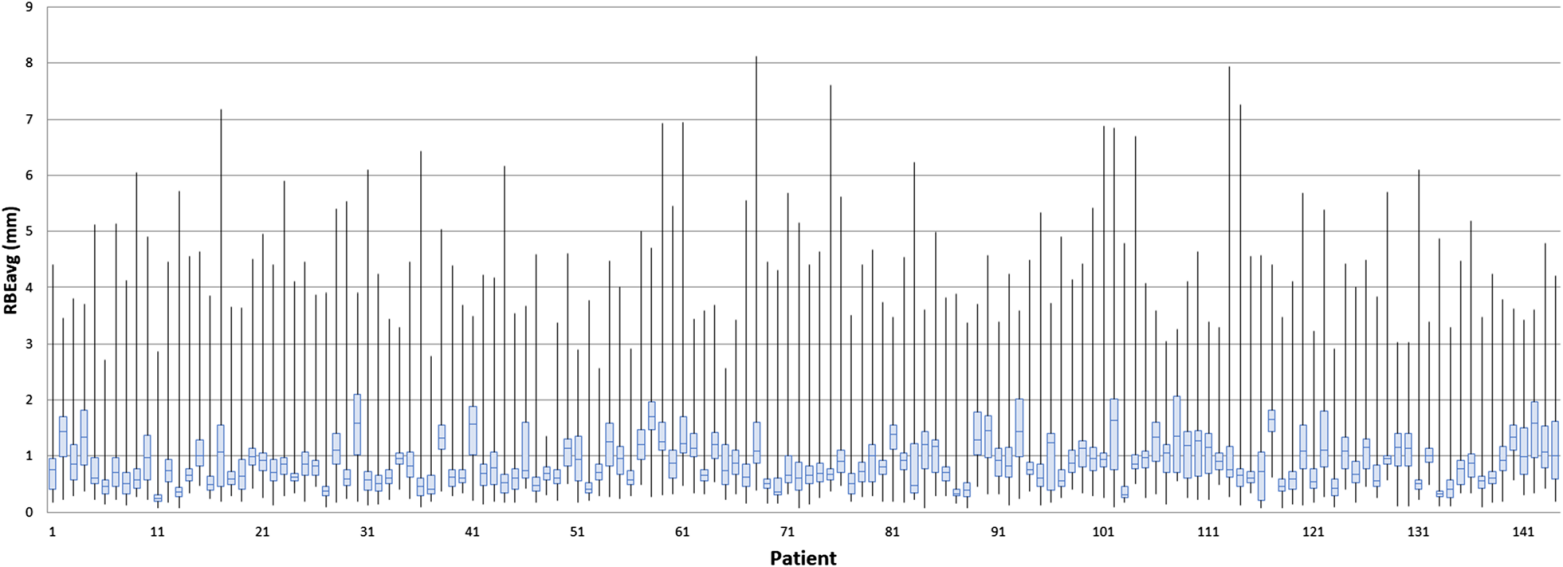
Box plot visualization of RBEavg observed over the whole treatment for all cases

In all, 13%, 17%, 13%, and 14% of RBEavg, RBEf1, RBEf2, and RBEf3 values, respectively, exceeded 1.5 mm during the treatment course. FM4 represents a special case, as 12% of the observed values exceeded the 1.5-mm threshold and an additional 16% of the values are missing (compared with the other FMs) because FM4 was not used for tracking due to its RBE being consistently > 2.5 mm (Fig. 2). Figure 3 shows box plot visualization of RBEavg observed over the whole treatment for all cases.

### Correlation

In an ideal case in which deformation does not exist, the RBE value will be zero because the position of the FMs will be constant. With this assumption, each pair of FMs should correlate completely.

The Pearson correlation coefficients were in the range 0.66–0.78 (*P* < 0.001) for all combinations of all FM RBEs in all patients. Lower coefficients were observed in every combination with FM4 (R = 0.66–0.69), which points to different deformation of the prostate apex. In comparison, the highest correlation (R = 0.78) was observed between FMs 2 and 3 implanted in the middle left and right portion of the prostate.

**Fig. 4:**
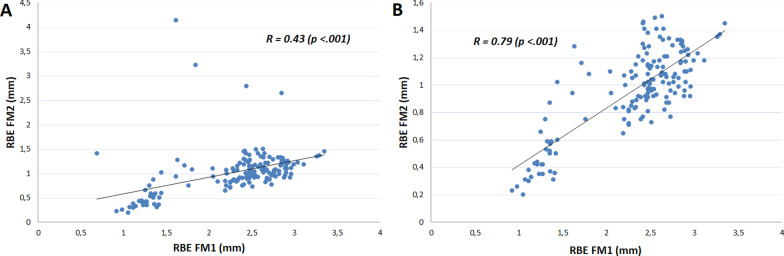
Dependence of the RBE of FM1 on the RBE of FM2 for a selected case. Panel **a** shows the correlation with outliers included; panel **b** shows the same data without outliers

If we look at the individual cases in more detail, the correlation is reduced due to outliers. Outliers represent the shift of an individual fiducial. Figure 4 shows an example of correlation between FM1 and FM2 during the whole treatment course, with and without outliers.

**Fig. 5:**
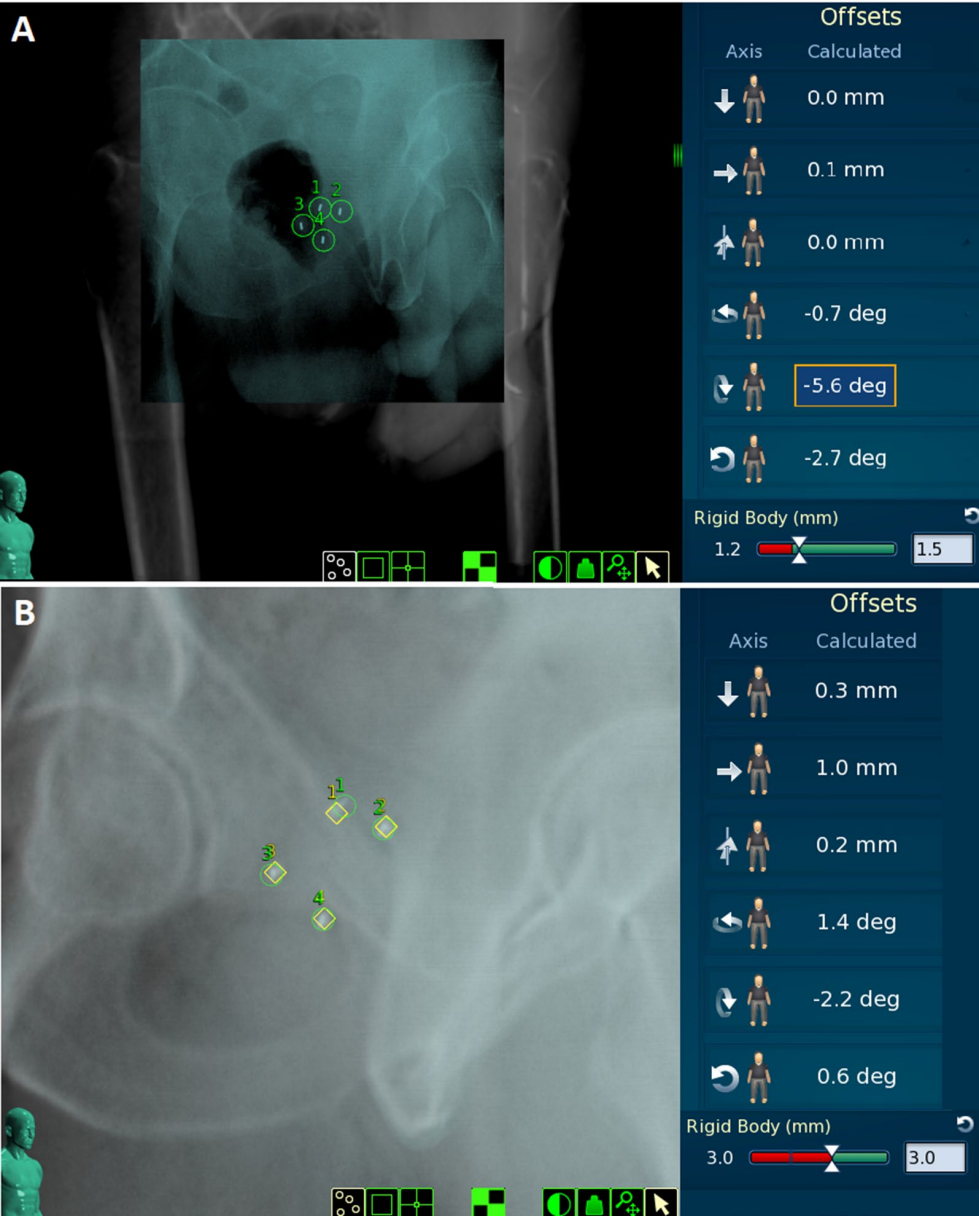
Impact of rectal and bladder filling on prostate position and deformation. Panel **a** shows gas in the rectum causing high-pitch rotation and interruption of the treatment while the RBE is below threshold. Panel **b** shows a comparison of 4 FMs’ positions between the reference CT (rhombus) and during treatment (circle), with the translational and rotational offsets and the highest detected rigid body error (in FM1)

**Fig. 6:**
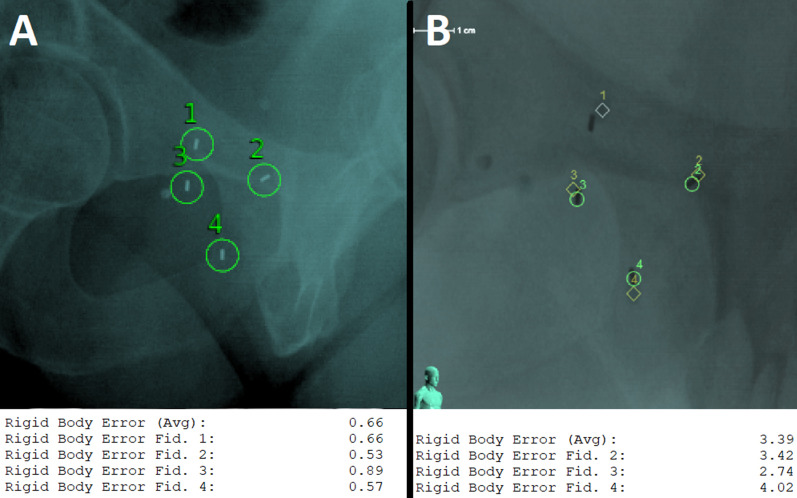
Example of prostate deformation resulting in high RBE values due to filling of the rectum. Panel **a** shows the positions of the 4 FMs and their RBE values during the first fraction. Panel **b** shows the FM positions and RBE values during the fourth fraction, when the patient did not empty his rectum before the treatment (FM1 was disabled)

From our experience, the rectum filling with any mass causes more deformation than does gas, which is associated with high-pitch (head up) rotation of the prostate. Similarly, substantial differences in bladder filling frequently cause deformation of the superior part of prostate. The next figures demonstrate 3 exemplary cases. Figure 5 (panel a) shows gas in the rectum causing high-pitch rotation and interruption of the treatment while RBE is below threshold. Panel b represents a scenario of poor bladder filling. The patient forgot to drink water in advance, then emptied his rectum and immediately drink 0.5 L of water just a few minutes before the start of the treatment. The FM1 reported an RBE of 3 mm. To be able to start treatment using all the FMs, we waited 20 min to let the bladder fill properly, which resolved the high RBE of FM 1. Figure 6 shows the worst case in terms of prostate deformation. Panel a shows the FMs and RBE values during the first fraction. The patient did not empty his rectum for the fourth fraction, resulting in high RBEs for all FMs.

## Discussion

In this study we evaluated the deformation of the prostate during hypofractionated stereotactic body radiation therapy, using the rigid body error of implanted fiducial markers. The use of FMs in prostate treatment has expanded rapidly in recent years and offers quick and precise target detection. Several studies have described prostate intrafraction translation and rotation [3–8]. based on different techniques. However, limited information about prostate deformation is available in the literature [9–14]. The mentioned studies differ in the number of included patients (range 8–56) and methodology. Most of them evaluate changes in the prostate surface using repeated CT scans over the treatment session [9, 12], cone-beam CT (CBCT) before treatment [11], or randomly assigned MRI [10, 14]. Kupelian et al. [13] measured intermarker distances using orthogonal X-rays before the start of each fraction. Xie at [20] reported the characteristics of intrafraction prostate motion in 21 patients during hypofractionated radiotherapy with the CyberKnife and briefly evaluated prostate deformation by using the RBE values of 4 patients over a limited time. To our knowledge, ours is the first study to show the rigid body error of implanted FMs in a large sample of patients during the whole course of treatment. All selected patients had 4 FMs implanted in the precise geometry required for efficient tracking.

The average RBE of all FMs was 0.93 ± 0.39 mm, indicating that overall deformation was small. Moreover, values below the system algorithm threshold of 1.5 mm ensured that the targeting error due to rigid body deformation was below 1 mm. This result is similar to that of Deurloo et al. [12], who reported no significant variations in gross tumor volume in relation to shape. Wielen et al. [9] rigidly registered fiducial markers within the prostate, comparing 3 repeated CT scans with pretreatment CT, and found that the deformation of the prostate relative to the FMs was small (SD < 1 mm). Apparently, the prostate surface moves along with the FMs as an almost fully rigid body.

Nakazawa et al. [11] obtained 7 repeated CBCT scans during the course of treatment. The mean prostate deformation was 0.6 ± 1.7 mm, a fourfold higher deviation than we observed. The average deformation differed among the manually defined segments. Unlike the results of Wielen et al. [9], their analysis revealed significant correlation between anterior–posterior prostate CoG displacement and deformation in the middle-anterior and middle-posterior segments. They reported a maximum deformation of 13 mm, which is similar to our maximum detected value of 11.3 mm. Even though we reported similar mean RBE values for individual markers placed in different segments, lower correlation coefficients were observed in every combination with FM4, which points to different deformation of the prostate apex. In comparison, the highest correlation was observed between FMs 2 and 3, which were implanted in the middle left and right portion, respectively, of the prostate.

Xie et al. [20] evaluated RBE from CyberKnife treatment in 4 patients, and it was generally below 1.5 mm. They reported correlation coefficients close to 100% among three fiducials (separated into pairs), which is not in accord with our results. The Pearson correlation coefficients for all combinations of FM RBEs in all our patients were in the range 0.66–0.78. Lower coefficients were observed in every combination with FM4, which points to different deformation of the prostate apex. Detailed analysis showed that overall correlation was affected by shifts of one FM relative to the others.

Kupelian et al. [13] observed deformation of more than 5 mm in 9% of patients and reported the average absolute variation (equivalent of RBE) of all markers to be 1.01 ± 1.03 mm. This deviation is more than twofold higher than what we observed. As they acquired X-ray images for analysis only before the start of the treatment, the results are limited to interfraction variation compared with our average of 34 X-ray acquisitions per fraction (intrafraction variation).

All patients were instructed to use the same preparation protocol from the planning CT to the last fraction. They were instructed to empty their rectum and drink 0.5 L of water 1 h before the examination. In spite of this, during treatment we frequently saw gas in the rectum causing rotations of the prostate but, as a rule, deformation was not affected. Kupelian et al. [13] did a detailed review of cases where FMs changed their relative positions frequently. That review revealed that marker mobility was caused by prostate deformation secondary to rectal filling. In contrast, Nichol et al. [10] reported that prostate deformation is unrelated to differential bladder and bowel filling. From our experience, rectal filling with any solid mass causes more deformation than does gas. Similarly, a substantial change in bladder filling frequently causes deformation of the superior part of the prostate. This finding is in accord with that of Kerkhof et al. [14], who reported a mean deformation of the posterior side of the prostate of 3.7 ± 2.3 mm in volunteers at maximum rectal volume compared with an empty rectum. All these cases represent situations where patient preparation has to be improved before the treatment begins or it becomes necessary to interrupt the treatment for a while to keep the RBE below a strict threshold.

Nichol et al. [10] evaluated patients who underwent conventional therapy in 42 fractions. They reported decreases in prostate volume of 0.5%/fraction and migration of FMs by 0.05 mm/fraction. Similarly Shirato et al. [22] showed that distances among the three markers gradually decreased during RT, with the mean gradient of the regression coefficient equal to − 0.053 mm/day. We observed that RBEavg was significantly lower in the first fraction, which is consistent with the hypothesis of prostate shrinkage during radiotherapy. However, RBE values are not directional, systematic change in FMs configuration over time indicates a systematic change in prostate shape throughout the treatment (Fig. 1). Moreover, in 15% of cases it was not possible to use FM4 for the fifth fraction due to its RBE > 2.5 mm. FM4 is located closest to the rectum. Proctitis and associated changes in peristalsis progressed during radiotherapy what probably affected the deformation of this part of the prostate.

This study describes the possible range of prostate deformation, based on RBE values. RBE values are not directional (only absolute differences between the reference position and positions during treatment were evaluated), and this can be considered a limitation of the results. Only values from the treatment after initial patient alignment were included in the study.

The CyberKnife treatment time per fraction is longer compared with conventional systems, which allows more detailed observation of target motion. Online tracking guarantees treatment interruption when prostate deviation from reference alignment is above the threshold necessary for precise and safe dose delivery. Our observations indicate that prostate deformation can be independent of rotation (Fig. 5, panel a) and significant in only one prostate segment (Fig. 5, panel b) or in the whole volume (Fig. 6). Fast treatment delivery may be advantageous but carries a risk in the event of sudden large undetected deformations.

## Conclusions

In ideal conditions, prostate deformation is generally small but very sensitive to rectal and bladder filling. We observed RBE up to 11.3 mm. The overall correlation between FMs was affected by larger shifts of individual fiducials, indicating that the prostate is not a “rigid” organ. FM 4 in the prostate apex was not possible to use in 16% of observations, as the RBE was > 2.5 mm. Systematic change of RBE average between subsequent fractions indicates a systematic change in prostate shape.

## Data Availability

The datasets analysed during the current study are not publicly available because contains patient‘s identificators.

## Abbreviations

CBCT: Cone-beam CT
CoM: Center of mass
CoG: Center of gravity
CT: Computed tomography
DRR: Digitally reconstructed radiograph
FM: Fiducial marker
MRI: Magnetic resonance imaging
RBE: Rigid body error
RBEavg: Average rigid body error
SD: Standard deviation
